# Preclinical Investigation of a Lipoglycopeptide Dry Powder Inhalation Therapy for the Treatment of Pulmonary MRSA Infection

**DOI:** 10.3390/pharmaceutics15092250

**Published:** 2023-08-31

**Authors:** Donna M. Konicek, Adam J. Plaunt, Sachin Gharse, Sasha J. Rose, Arielle Dorfman, Amruta Sabnis, Thomas Baker, Helena Gauani, Donald Chun, Zhili Li, Walter R. Perkins, David Cipolla, Vladimir S. Malinin

**Affiliations:** Insmed Incorporated, Bridgewater, NJ 08807, USA

**Keywords:** DPI, antibiotics, cystic fibrosis, MRSA, lipoglycopeptide, inhalation

## Abstract

The increased prevalence of pulmonary methicillin-resistant *Staphylococcus aureus* (MRSA) infection in patients living with cystic fibrosis (CF) is concerning due to a correlation with reduced life expectancy and lack of available treatment options. RV94 is a next generation lipoglycopeptide designed for pulmonary delivery that preclinically demonstrated high potency against MRSA in planktonic and protected colonies and improved pulmonary clearance relative to same class molecules. Here, RV94 was formulated into a dry powder for inhalation (DPI) to investigate the localized treatment of pulmonary MRSA presented in a potentially more convenient dosage form. RV94 DPI was generated using a spray-drying process with 12.5 wt% trileucine and demonstrated aerosol characteristics (2.0 μm MMAD and 73% FPF) predictive of efficient pulmonary deposition. In vivo PK from a single dose of RV94 DPI delivered by inhalation to rats yielded lung levels (127 μg/g) much greater than the MRSA minimum inhibitory concentration (0.063 μg/mL), low systemic levels (0.1 μg/mL), and a lung t_1/2_ equal to 3.5 days. In a rat acute pulmonary MRSA model, a single dose of RV94 DPI delivered by inhalation either up to seven days prior to or 24 h after infection resulted in a statistically significant reduction in lung MRSA titer.

## 1. Introduction

Cystic fibrosis (CF) is a rare genetic disease caused by defective expression and function of the cystic fibrosis transmembrane conductance regulator. This genetic anomaly causes abnormal mucus secretions and acidification of the airway surface fluid which enhances patient susceptibility to chronic infection from opportunistic pathogens that colonize the airways [1,2]. Of rising concern is the increasing prevalence of chronic pulmonary methicillin-resistant *Staphylococcus aureus* (MRSA) infection which now affects one quarter of CF patients, has no approved therapies, and has been linked to greater incidences of morbidity and mortality [3,4]. Intravenous vancomycin, a potent glycopeptide antibiotic, is commonly used as front-line therapy for MRSA infection in the clinic. However, poor pulmonary penetration, dose-limiting renal toxicity, and the requirement to be administered on an inpatient basis in a hospital setting make it an inconvenient and ineffective solution to treat chronic MRSA respiratory infections in CF patients [5].

Antibiotic inhalation therapy, marked by the clinical success of the aerosolized aminoglycoside tobramycin used for the treatment of chronic *Pseudomonas aeruginosa* pulmonary infection, is a staple for the treatment of CF lung disease. Tobramycin inhalation therapy was initially administered as a nebulized solution [6] and then as a dry powder for inhalation (DPI) [7] and in both forms has demonstrated excellent performance owing to benefits of local delivery associated with efficient pulmonary deposition and reduced systemic exposure [8]. An analogous strategy for the treatment of chronic pulmonary MRSA infection in CF utilizing inhaled vancomycin was investigated clinically; however, when delivered off-label as a nebulized solution or as a DPI in a sponsored trial (NCT03181932), it failed to generate effective clinical outcomes [9,10]. It has been postulated that the clinical failure of vancomycin inhalation therapy could be tied to the growing body of research highlighting inefficient permeability of the compound towards biological membranes, which we and others have shown can protect MRSA colonies inside of cells and in biofilms [11,12,13,14] even when the drug is delivered locally and in high concentrations to the affected tissues [14].

**Figure 1:**
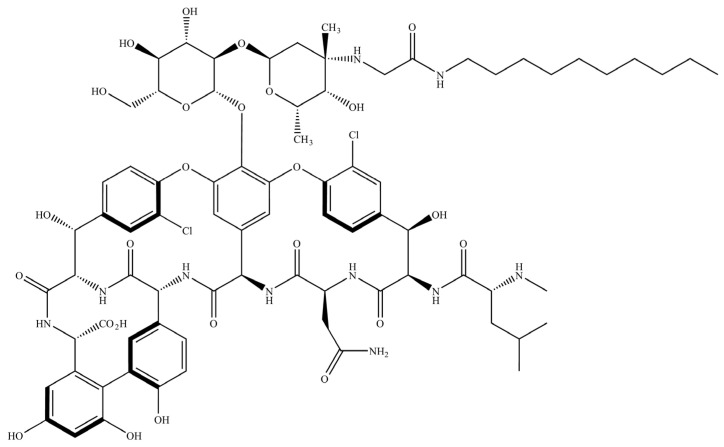
Chemical structure of RV94 (N^van^-2-amino-N-decylacetamide vancomycin).

Lipoglycopeptides, hydrophobic derivatives of glycopeptides, have demonstrated potent activity against MRSA owing to their multiple bactericidal mechanisms and have been reported to efficiently penetrate and kill bacteria in biofilms and cells [15,16,17]. Drawbacks to clinical lipoglycopeptide use involve poor elimination kinetics in vivo, the requirement to be administered parenterally in a hospital setting, and renal toxicity for certain compounds (e.g., telavancin) [18,19,20]. To improve upon these shortcomings, we synthesized and reported on a new subclass of next generation lipoglycopeptide compounds designed specifically for pulmonary delivery [13]. In doing so, evidence was provided to demonstrate that select compounds of this class could conserve the high antimicrobial potency and membrane-penetrating qualities of the traditional lipoglycopeptides while improving upon their metabolism and clearance in vivo. RV94 (N^van^-2-amino-N-decylacetamide vancomycin) (Figure 1), a hydrophobic derivative of vancomycin, was identified as a promising lead from this new subclass owing to (i) its superior in vitro and in vivo performance when benchmarked to vancomycin and (ii) its optimized PK profile after inhalation of a nebulized solution when compared to other compounds of its class.

The treatment burden for patients with CF is significant, requiring an estimated two to three hours per day of routine therapeutic maintenance, which underscores the challenge for advancement of new medicines into a CF treatment regimen [21]. To avoid unfavorably adding to this burden, we further formulated RV94 into a high dose DPI with the design goal being that the product could be conveniently stored and efficaciously dosed using a simple inhaler. Herein the formulation, physiochemical characterization, and preclinical activity of RV94 DPI are presented.

## 2. Materials and Methods

**Ethical Statement.** All the in vivo experiments were conducted according to the guidelines for animal ethics in accordance with the institutional animal care and use committees (IACUC) at Rutgers University (Protocol PROTO999900355, Piscataway, NJ, USA).

**Experimental Compounds.** RV94 synthesis was carried out by reductive amination between an aldehyde and the vancosamine nitrogen of vancomycin as previously described [13]. The compound was purified by preparative reverse phase high-performance liquid chromatography (HPLC, Waters Corporation, Milford, MA, USA) and isolated as a monolactate salt. Compound identity was confirmed using liquid chromatography mass spectrometry (LC-MS, Sciex, Framingham, MA, USA). The chemical purity of RV94 was measured using HPLC-UV and was >95%. Vancomycin HCl was sourced from Chemwerth Incorporated (Woodbridge, CT, USA).

**RV94 DPI Formulation and Spray-Drying.** Dry powders for inhalation (DPIs) were prepared using the Buchi B-290 spray dryer with Inert Loop B-295 condenser and B-296 dehumidifier (Buchi Labortechnik AG, Flawil, Switzerland). A spray-drying feed stock was prepared by dissolving RV94 monolactate and trileucine (87.5:12.5 wt%) in a 60:40 (*v/v*) n-propanol: water solvent system (Sigma, St. Louis, MO, USA). The pH of the feed stock was adjusted to 5.88, using sodium hydroxide (NaOH) at an NaOH:RV94 mole ratio of 0.4:1. The feed stock was then spray-dried using the following process parameters: inlet temperature 100 °C, outlet temperature 57 °C, feed flow rate 4.05 mL/min, feed concentration 15 mg/mL, and aspiration 100%. A secondary drying step was applied to the powder by storing it under vacuum oven overnight at room temperature.

**RV94 DPI Residual Moisture Content.** The moisture content of the spray-dried RV94 powder was measured using an Aquastar AQV33 Karl Fischer Titrator with an installed 5 mL burette (EMD-Millipore, Burlington, MA, USA). Approximately 30 mg of sample was weighed and transferred to the titration vessel. Moisture content was measured using the following parameters and materials: Aquastar Combi Titrant 2, 12, 1% NIST water standard (Sigma, St. Louis, MO, USA), Aquastar CombiCoulomat fritless Karl Fischer reagent (EMD-Millipore, Burlington, MA, USA), drift < 50 µg/min, potential range 80–100 mV, and mix time was 300 s.

**Scanning Electron Microscopy (SEM).** The morphology of the spray-dried powder was analyzed using SEM imaging (Rutgers University Materials Science and Engineering Core Facility). Sample powders were poured on a carbon tape and then coated with 20 nm gold (Au) using an EMS150T ES sputter coater (Electron Microscopy Sciences, Hatfield, PA, USA). Field emission-scanning electron microscopy (FE-SEM) was used to observe the particle morphologies, using a Zeiss-Sigma FE-SEM (Zeiss, Oberkochen, Germany) with an operating voltage of 5 keV. The working distance was kept between 8 and 10 mm to obtain optimized resolution.

**Particle Size Distribution Determined by Laser Diffraction.** Laser diffraction was employed to measure particle size distribution in the dry powders, using Sympatec HELOS/BR unit equipped with an ASPIROS feeder and a RODOS/M dry powder dispersing unit (Sympatec GmbH, Clausthal-Zellerfeld, Germany). Approximately 5 mg of the dry powder was added to the sample vial, which was then inserted into the ASPIROS feeder. The sample was measured at a measuring range R1 with a RODOS standard trigger at a primary pressure of 0.5 bar and feed velocity of 15 mm/s.

**In Vitro Aerosol Characterization.** Aerodynamic particle size distribution (APSD) characterization was performed using the next generation impactor (NGI) (MSP Corp., Shoreview, MN, USA) assembled in line with the USP (United States Pharmacopeia, North Bethesda, MD, USA) induction port, pre-separator, filter assembly, and placed into an environmental control chamber equilibrated to 23 °C and 35% relative humidity. Approximately 10 mg of RV94 DPI was added to a size 3 HPMC capsule (Qualicaps Inc., Whitsett, NC, USA), and the capsule was loaded into the monodose RS01 high-resistance dry powder inhaler (Plastiape S.p.A., Osnago, Italy). The loaded device was then inserted into the USP induction port via an adapter and a vacuum pump along with a TPK 2100 critical flow controller (MSP Corp., Shoreview, MN, USA) to maintain the desired flow rate of 60 L/min for 4 s to aerosolize the dry powder. Following deposition of aerosol within the NGI, aerosol recovery from each component was performed by adding a specified volume of solvent to the NGI collection cups (5 mL), induction port and adapter (10 mL), pre-separator (10 mL), filter (5 mL), device (10 mL), and aerosolized capsule (10 mL). The mass of RV94 associated with each component was quantified using an HPLC system and charged aerosol detector (CAD) (Thermo Fisher Scientific, Waltham, MA, USA) on a C18 column. The emitted dose represents the mass of RV94 that exited the device and was recovered from the NGI components. The aerosol MMAD was calculated in accordance with compendia [22,23] and utilizing cutoff diameters of 8.06, 4.46, 2.82, 1.66, 0.94, 0.55, and 0.34 µm for stages 1 to 7, respectively. The FPF represents the proportion of RV94 that is present in particles with an aerodynamic diameter less than 5 µm and is expressed relative to the total emitted dose.

**In Vitro Susceptibility.** Clinical and Laboratory Standards Institute (CLSI) guidelines were followed for the determination of the minimum inhibitory concentration (MIC), using cation-adjusted Mueller Hinton broth microdilution at a starting inoculum level of 5 × 10^5^ colony forming units (CFUs) per mL [24,25]. MIC was read by optical density on a BioTek Synergy H1 plate reader (BioTek, Winooski, VT, USA) at 595 nm. Strains used were MRSA ATCC BAA-1556 (USA300, SCC*mec*IV, pvl^+^) and MSSA ATCC 29213 (American Type Culture Collection, Manassas, VA, USA). MIC data are reported as a range to reflect the fact that the value is determined using step dilution.

**In Vivo PK.** Male Sprague Dawley rats (Charles River Laboratories, Senneville, QC, Canada) that weighed between 245 and 325 g were used to investigate PK of single dose administrations of RV94 DPI, using a 12-port nose-only inhalation tower (CH Technologies, Westwood, NJ, USA). Animals were acclimated for approximately 1 week prior to the study start. Rats were loaded into the chamber in cohorts of ten at the time of dosing. RV94 DPI was aerosolized using the Vilnius Aerosol Generator (VAG) dry powder dispenser (CH Technologies, Westwood, NJ, USA) at output rates ranging from 6 to 15 mg/min. Additional airflow, from dry compressed air, was provided to the VAG at a flow rate of 8 L/min to support aerosolization of the DPI. Single dose administrations of RV94 DPI were delivered to the animals at a drug concentration of 875 mg/g to deliver a target dose of approximately 10 mg/kg. The remaining ports on the chamber were fit with an aerosol sampling filter for dose determination and the Marple cascade impactor, NS-298 (National Institute for Occupational Safety and Health, Washington, DC, USA) for APSD characterization. The cascade impactor was set to a specified flow rate and collection time designed to eliminate stage carry over and filter clogging. The average dose delivered to the nose of each animal was calculated using an algorithm that was previously described [26] and was based on the analytical quantification of drug product deposited on the aerosol sampling filter. Three cohorts were used to accommodate an *n* = 5 for immediately post-dose groups (approximately 30 min after completion of dosing) and *n* = 3 for all other timepoints. Animals were euthanized for collection of whole lungs and plasma at timepoints ranging from immediately post-dose (0.5 h after conclusion of aerosol delivery) to 21 days. RV94 was extracted from tissues, using a protein precipitation method whereby the lung tissue or plasma was homogenized in a mixture containing acetonitrile (ACN) (3:1 ACN: homogenate *v*/*v*) and an analogue lipoglycopeptide compound that was custom made used as an internal standard. Extracted samples were analyzed by tandem mass spectrometry (LC-MS/MS) (Sciex API4500 or API6500+ mass spectrometer (Sciex, Framingham, MA, USA)).

**In Vivo Efficacy.** Male Sprague Dawley rats (Envigo, Indianapolis, IN, USA) weighing between 225 and 250 g were acclimated to the study facility as described above in the PK methods section. Neutropenia was induced with intraperitoneal cyclophosphamide (Sigma, St. Louis, MO, USA) on Day-4 (150 mg/kg) and Day-1 (100 mg/kg) relative to infection. On Day 0 at 0 h, rats were challenged with MRSA ATCC BAA-1556 (USA300) (American Type Culture Collection, Manassas, VA, USA) by intranasal instillation at a target titer of 8.0–8.3 Log_10_ CFU, which was determined using an optical density measurement and back-counting inoculum CFU count. For inoculum preparation, a frozen aliquot MRSA ATCC BAA-1556 stored at −80 °C was thawed, transferred into tryptic soy broth (TSB, Sigma, St. Louis, MO, USA), and incubated at 37 °C while shaking for 16 h. Cultures were centrifuged at 2000× *g* for 5 min, and the pellet was resuspended in PBS. For the in vivo challenge procedure, rats were anesthetized using 2–5% isoflurane in 100% oxygen, then restrained with a scruff grip. A volume equal to 200 μL of inoculum was introduced to one nare, and the rat was held in a vertical position until the challenge was completely aspirated. Rats were monitored following the challenge to ensure normal respiration and then returned to their cage.

For the pretreatment dosing paradigm, rats (*n* = 10 per cohort) were treated with either air sham control (dry compressed air at a flow rate of 8 L/min) or with RV94 DPI, using procedures detailed in the PK section for nose-only inhalation at a target nose dose of 10 mg/kg on Day-7 and Day-1 relative to bacterial challenge. For the therapeutic (after infection) dosing paradigm, rats (*n* = 10 per cohort) were treated with RV94 DPI or air sham at 24 h after infection. In both dosing paradigms, the treatment and control groups were held in the inhalation chamber for a total of 30 min to account for stress effects associated with restraint. All groups underwent necropsy at 48 h after infection for enumeration of pulmonary MRSA titer. Animals were euthanized using carbon dioxide overexposure. Whole lungs were aseptically removed, and lung weights were recorded. Lungs were homogenized using 3.2 mm stainless steel beads in sterile water with a Bead Ruptor Elite homogenizer (Omni International, Kennesaw, GA, USA). Mixed, homogenized samples were serially diluted and plated on Vogel–Johnson *S. aureus* selective media (Sigma, St. Louis, MO, USA), using sterile disposable cell spreaders. Plates were then incubated at 37 °C for 24–48 h before colonies were enumerated.

**Statistical Analysis.** Graph Pad Prism 9.3 (La Jolla, CA, USA) was used for statistical analyses and data plotting. Area under the curve (AUC) was determined using the trapezoidal method [27]. A one-phase exponential decay was used to fit the PK data for the determination of lung and plasma half-life and plateau. In vivo efficacy statistical analysis was based on the Mann–Whitney test.

## 3. Results

### 3.1. RV94 DPI Formulation and Physiochemical Characterization

RV94 DPI was generated using a spray-drying process whereby RV94 monolactate and trileucine were dissolved in a solvent system containing 60:40 (*v*/*v*) n-propanol: water. The residual moisture content of the powder after spray-drying was 3.5% as determined by Karl Fischer titration. The formulation used in this study was composed of 87.5% RV94 and 12.5% trileucine by weight. This composition provided a high drug content while demonstrating good and aerosol performance and stability for in vivo investigation. The average process yield for the manufacture of RV94 DPI was approximately 65% (*n* = 3). Analytical measurements of drug and excipient concentrations of the spray-dried product were nearly equivalent to the concentration of process inputs for three independent spray-dried batches (±2.4%), indicating uniform distribution of the components within the collected particles and thermal stability during the spray-drying process. The surface composition of RV94 DPI was characterized by X-ray photoelectron spectroscopy (XPS) (Table S1) and the results show a trileucine concentration that is greater than the theoretical proportional content in the spray-drying feedstock (32.3% measured vs. 12.5% theoretical), indicating enrichment of the excipient on the surface of the powder.

**Figure 2:**
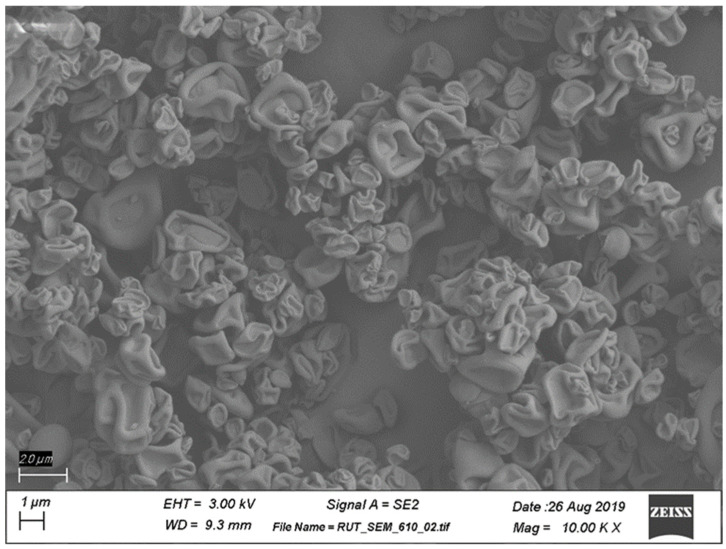
SEM of RV94 DPI containing 12.5 wt% of trileucine. Scale bar = 2 μm.

RV94 DPI morphology was investigated using scanning electron microscopy (SEM). Corrugated particles were observed as shown in the micrograph in Figure 2. RV94 DPI mean geometric particle size (d50) determined by laser diffraction ranged was 2.8 μm. The particle size distribution was observed to be unimodal and did not change over the course of a month when stored under ambient conditions (Figure S1). The RV94 DPI X-ray powder diffractograms in Figure S2 also point to the stability of the formulation, demonstrating a pattern of non-crystallinity which indicates amorphous solid-state character that is retained over the course of a month. The in vitro aerosol properties of the powder were investigated using a next generation impactor (NGI) and yielded a mass median aerodynamic diameter (MMAD) equal to 2.0 µm and a fine particle fraction (FPF, a determinate of the respirable dose based on a particle size cutoff of <5 μm) equal to 73% (Table 1). NGI stage deposition of the emitted dose of RV94 DPI is shown in Figure S3.

### 3.2. In Vitro Activity

The in vitro antibacterial activity of RV94 DPI was assessed via the measurement of minimum inhibitory concentration (MIC) against methicillin-sensitive and methicillin-resistant *S. aureus*, and it was compared to the unformulated compound and the parent molecule vancomycin (Table 2). The activity of RV94 DPI falls within an activity range that is comparable to the unformulated RV94 with an MIC equal to 0.063 μg/mL. Both the RV94 DPI and unformulated RV94 are significantly more potent (ca. 15-fold) than vancomycin against the methicillin-sensitive *Staphylococcus aureus* MSSA and MRSA isolates evaluated.

### 3.3. In Vivo Pharmacokinetics

**Figure 3:**
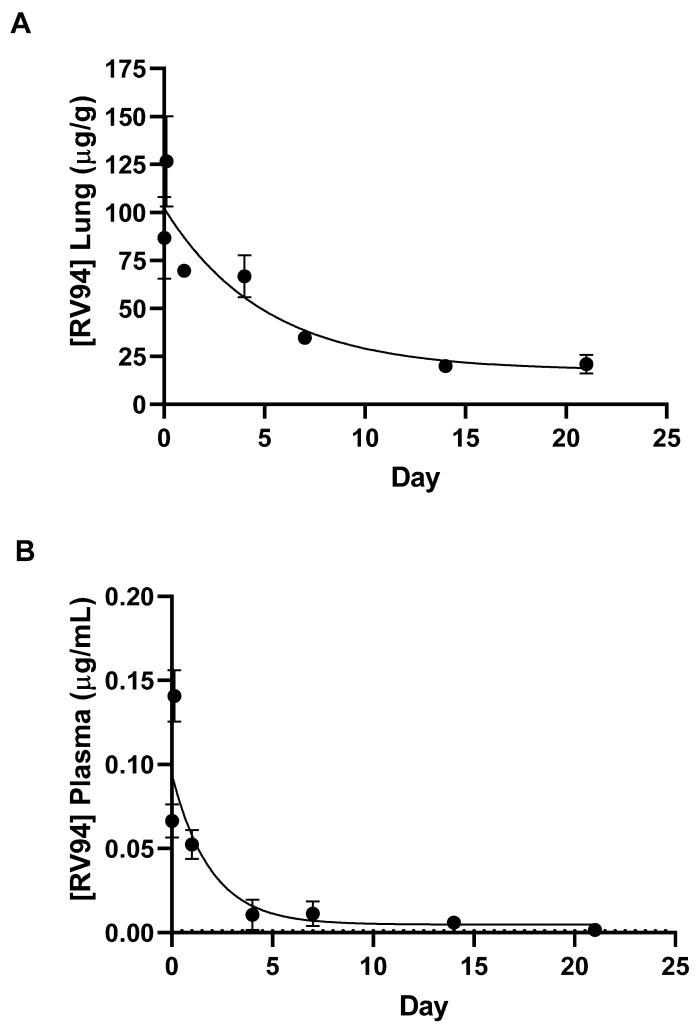
RV94 lung (**A**) and plasma (**B**) concentration following a single dose of RV94 DPI administered by nose-only inhalation to healthy rats using a VAG dry powder dispenser. Rats in *n* = 3 cohorts of *n* = 10 per cohort were dosed for 20 min, and the average mass of drug product aerosolized was 133 ± 14 mg (equivalent to 116 mg RV94 drug substance). The average RV94 dose delivered at the nose as determined by an inline sampling filter was 4.0 ± 0.6 mg/kg. Data were plotted as mean. Error is SEM; *n* = 5 for immediately post-dose, and *n* = 3 for all other timepoints. LOD for lung based on the lowest standard curve standard was 0.3 μg/g. LOQ = 0.0015 μg/mL for plasma. Data were fit using a one-phase exponential decay in GraphPad Prism 9.3. R^2^ = 0.85 for lung RV94 and 0.68 for plasma RV94.

A single dose nose-only inhalation in vivo pharmacokinetic (PK) evaluation of RV94 DPI was conducted using a Vilnius Aerosol Generator (VAG) dry powder dispenser to deliver the drug product to rats in a 12-port nose-only inhalation tower. Rats in *n* = 3 cohorts were dosed for 20 min, and the average mass of drug product delivered was 133 ± 14 mg (equivalent to 116 mg RV94). The average dose delivered at the nose as determined by the sampling filter was 4.0 ± 0.6 mg/kg RV94. The RV94 lung homogenate and plasma PK results for a 21-day collection period after dose are shown in Figure 3, and the calculated PK parameters are shown in Table 3. The average concentration of RV94 measured in the lung immediately post-dose, 0.5 h following conclusion of aerosol delivery, was 86.7 μg/g (Figure 3A). Cmax at 3 h post-dose and was equal to 127 μg/g (Figure 3A). This level was reduced to 69.7 μg/g by Day 1, demonstrating a 45% reduction over the first 24 h (Figure 3A). The calculated half-life of RV94 in the lung when delivered as a DPI was 3.5 days. Plasma Cmax was 0.14 μg/mL at 3 h post-dose (Figure 3B) or 0.1% of lung Cmax at the same timepoint, demonstrating markedly low systemic levels when given in a single dose by inhalation. The RV94 AUC_0–21Day_ was 787.7 μg*day/g, and the plasma AUC_0–21Day_ was 0.31 μg*day/mL yielding an AUC ratio of approximately 2500:1 lung: plasma (Table 3).

### 3.4. In Vivo Efficacy

**Figure 4:**
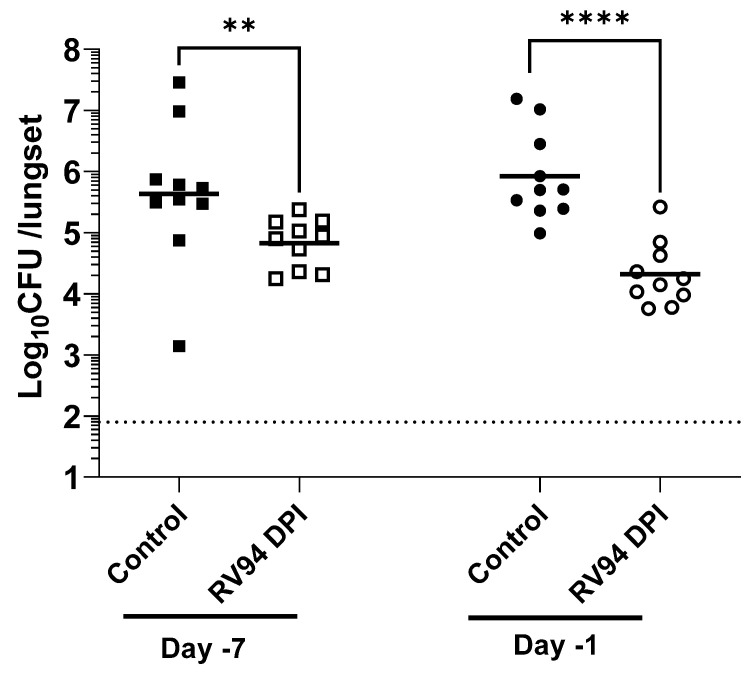
A single dose of RV94 DPI administered 7 days or 1 day prior to challenge demonstrates reductions in lung MRSA titer vs. control in an acute pulmonary MRSA (ATCC BAA 1556; USA300) infection in neutropenic rats. The estimated nose dose of RV94 measured from the sampling filter administered on Day-7 was 3.5 mg/kg and on Day-1 was 5.1 mg/kg. Lungs were excised for CFU enumeration at 48 h after infection. Data were plotted as geometric mean. *n* = 10 for all groups. LOD = 1.9 Log_10_CFU/lungset. Statistics are based on Mann–Whitney test; *p* = 0.009 for Day-7 treatment vs. air sham control, *p* < 0.0001 for Day-1 treatment vs. air sham control. ** Refers to *p* < 0.01 and **** to *p* < 0.0001.

**Figure 5:**
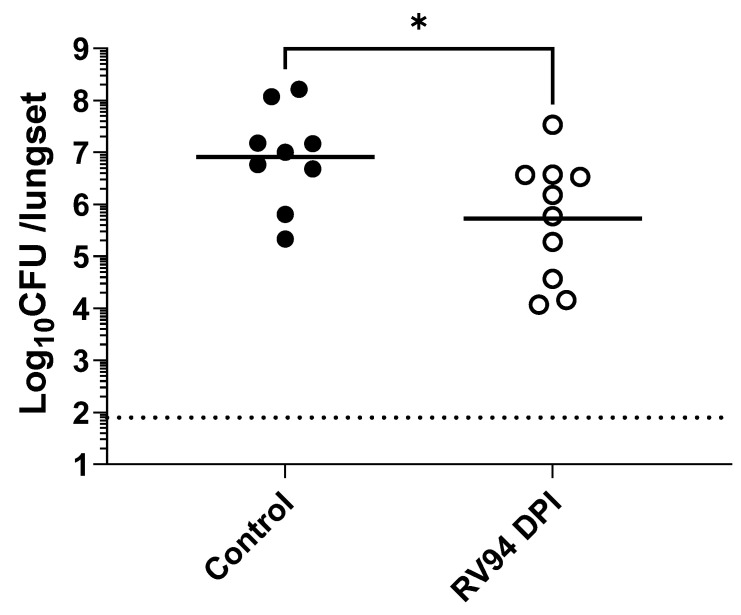
A single dose of RV94 inhalation powder administered 24 h after infection demonstrates a reduction in lung MRSA titer vs. control in an acute pulmonary MRSA (ATCC BAA 1556; USA300) infection in neutropenic rats. The estimated nose dose of RV94 measured from the sampling filter was 8.3 mg/kg. Treatment was administered 24 h after infection, and lungs were excised for CFU enumeration at 48 h after infection. Data were plotted as geometric mean. *n* = 9 for control and 10 for RV94 DPI. In the control group, one rat succumbed to the infection and died. LOD = 1.9 Log_10_CFU/lungset. Statistics are based on Mann–Whitney test; *p* = 0.02. * Refers to *p* < 0.05.

RV94 DPI in vivo efficacy was evaluated in a rat acute pulmonary MRSA (ATCC BAA-1556; USA300) infection model when delivered by nose only inhalation using a VAG aerosol generator as described in the PK section. First, a pretreatment paradigm was evaluated where a single dose of RV94 DPI was delivered to rats seven days or one day prior to intranasal MRSA challenge, and lungs were excised for CFU enumeration at 48 h after infection. The data summarized in Figure 4 show an average reduction of 0.8 Log_10_CFU/lungset in pulmonary MRSA titer when the therapeutic was delivered seven days prior to bacterial challenge and 1.6 Log_10_CFU/lungset when delivered one day prior to challenge. In both cases, the treatment effect was statistically significant when compared to rats that received air sham (*p* = 0.009 for Day-7, and *p* < 0.0001 for Day-1; Mann–Whitney test). While the observed therapeutic effect was even greater when RV94 DPI treatment was administered one day prior to challenge, the measured delivered dose was also nominally higher on that day than that in Day-7 pre-dose group (5.1 vs. 3.5 mg/kg), which may factor into this observation. Next, the in vivo efficacy of RV94 DPI was investigated using a therapeutic dosing paradigm, where the drug product was administered as a single dose 24 h after bacterial challenge in an acute pulmonary MRSA infection model in rats using nose-only inhalation. As shown in Figure 5, the treatment group demonstrated an average reduction of 1.2 Log_10_CFU/lungset in pulmonary MRSA titer when compared to air sham control, and the delivered dose was 8.3 mg/kg. This effect was statistically significant when compared to the control (*p* = 0.02; Mann–Whitney test).

## 4. Discussion

We previously reported that RV94, a next generation lipoglycopeptide, has potent antibacterial activity that is superior to vancomycin in vitro against planktonic, intracellular, and biofilm MRSA and in vivo in an acute pulmonary MRSA infection in rats when delivered by nose-only inhalation as a nebulized preparation [13]. Additionally, we showed that RV94 possesses a pulmonary elimination kinetics advantage over compounds of its class when delivered by inhalation to rats, which is significant since in vivo accumulation is reported to be a barrier to lipoglycopeptide development [13,19]. Given the unmet need for the treatment of chronic pulmonary MRSA infection in CF in combination with the significant treatment burden experienced by the typical patient with CF, we sought to convert the inhaled formulation of RV94 into a high dose DPI [3,4,21].

A DPI product of RV94 was generated using an optimized spray-drying process and formulation that demonstrated good batch-to-batch repeatability, recovery, physicochemical stability (Figures S1 and S2), and aerosol performance (Table 1). A feedstock co-solvent consisting of 60:40 n-propanol: water (*v*/*v*) enabled a suitable solubility profile for mixture of the drug substance RV94 monolactate with the tripeptide Leu-Leu-Leu (trileucine) in the concentration range of 10–15 mg/mL. Trileucine, included in the RV94 DPI at a mass ratio range of 12.5% relative to the drug substance (87.5%), was selected as a formulation excipient based on the precedent literature citing its effectiveness for drug stabilization during spray-drying and its benefit for aerosol performance [28,29]. XPS was used to characterize the surface composition of the powder and demonstrated that trileucine was measured at a higher concentration than the theoretical weight ratio of the excipient, which indicates enrichment at the surface of the powder, a desirable characteristic for product performance (Table S1). The observed morphology of RV94 DPI (Figure 2) via SEM imaging is consistent with a corrugated surface structure that has been reported for pharmaceutical DPI formulations containing trileucine as the primary excipient [28]. The in vitro aerosol characteristics of RV94 DPI measured by laser diffraction (Figure S1) and the next generation impactor studies (Figure S3) indicate the potential for efficient pulmonary deposition marked by an average MMAD of approximately 2 μm and fine particle fraction greater than 70% (Table 1).

To confirm the preservation of RV94 biological activity after spray-drying, RV94 DPI MIC against methicillin-susceptible and methicillin-resistant *S. aureus* were measured and compared to the unformulated compound and the parent molecule vancomycin (Table 2). The antibacterial activity of RV94 DPI fell within the range measured for the unformulated drug (0.063 μg/mL) against both isolates, demonstrating that the spray-drying formulation process and formulation itself did not negatively impact the potency of the compound. Notably this DPI product shows a marked antibacterial advantage over vancomycin, which is 15-fold less potent than RV94 DPI when evaluated against the same *S. aureus* isolates.

Single dose nose-only inhalation in vivo PK of RV94 DPI delivered to healthy rats revealed that initial drug levels in the lung were on the order of 2000-fold greater than MRSA MIC values after an average inhaled dose equal to 4 mg/kg was delivered to the animals. Lung drug levels were reduced to 45% of the Cmax one day post-dose and then to 15% of the Cmax over the three-week monitoring period yet remained significantly above the MIC, pointing to the potential durability of the therapeutic to combat chronic pulmonary MRSA infection. Furthermore, Cmax plasma drug levels (also occurring 3 h post-dose) were 0.1% of lung Cmax, demonstrating that inhaled RV94 DPI results in markedly low systemic levels when given in a single dose by inhalation which could translate to a low probability for harmful off-target side effects such as renal toxicity that have been observed with parenteral lipoglycopeptide therapy [18,20].

The calculated half-life of RV94 in the lung was 3.5 days when delivered as a DPI, demonstrating moderately improved pulmonary elimination kinetics when compared to a nebulized form of the same molecule which was previously reported to be approximately 4.5 days [13]. Since the RV94 drug substance is poorly soluble at physiological pH and must be prepared at pH > 9 to dissolve, a working hypothesis for this observation could be better dispersion and/or dissolution of the lipoglycopeptide in the pulmonary milieu when formulated as a dry powder. The SEM images of the RV94 DPI formulation show corrugated particles which would have greater surface area than that of a comparable mass of RV94 in a spherical particle. Furthermore, after initial wetting in the airway fluid there may be additional conversion to smaller drug particles for the DPI formulation than for the nebulized formulation which may precipitate on contact with the airway fluid. Both considerations favor the greater surface area for the DPI formulation when compared to the nebulized form which could explain improved dissolution in the lung lining fluid after inhalation of the aerosol [30].

The proposed mechanism for pulmonary elimination is mucociliary clearance since measured plasma levels at all timepoints were markedly low (Table 3) and lung and plasma levels of the primary metabolite were near or below the limit of quantitation. Notably, both the nebulized and DPI-formulated forms of RV94 demonstrated improved pulmonary elimination kinetics in rats when compared to other compounds of its class, including nebulized telavancin (t_1/2_ ca. 21 days), which underscores the value of the underlying molecular design that was customized for this route of administration [13]. In summary, the high local concentration of RV94 in the lung and low systemic levels meet the design hypothesis for the treatment of chronic pulmonary MRSA infection. Furthermore, the clearance trends could extrapolate to a recommendation for infrequent dosing, which would reduce the treatment burden and the potential for unwanted side effects in the target patient population.

RV94 DPI in vivo efficacy was evaluated in an acute pulmonary MRSA (USA300) infection in neutropenic rats, using dosing regimens that investigated antibacterial performance both before and after intranasal bacterial challenge. While chronic pulmonary infections are the proposed target indication for RV94 DPI, development of a reliable chronic MRSA rodent model has been challenging in part due to the steep mortality curve of this pathogen [31,32]. As such, we and others in this field have resorted to using acute pulmonary infection models to establish in vivo proof of concept [13,16,32,33,34].

The objective of the pretreatment experiment was to investigate whether RV94 DPI therapy remains biologically active during its residence time in the lung. A single dose of RV94 DPI was administered by nose-only inhalation seven days or one day prior to intranasal MRSA challenge. The data summarized in Figure 4 demonstrate a statistically significant reduction in pulmonary MRSA titer in the groups that received RV94 DPI therapy in comparison to the control animals that received air sham when dosing occurred up to seven days prior to challenge (*p* = 0.009 for Day-7, and *p* < 0.0001 for Day-1; Mann–Whitney test). These data confirm antibacterial activity of the drug throughout its duration in the lung. Notably, the observed therapeutic effect was even greater when RV94 DPI treatment was administered one day prior to challenge (1.6 Log_10_CFU/lungset reduction in titer for Day-1 versus 0.8 Log_10_CFU/lungset for Day-7). However, the measured delivered dose on the sampling filter was also nominally higher for Day-1 pre-dose group than for Day-7 pre-dose group (5.1 mg/kg for Day-1 vs. 3.5 mg/kg for Day-7), which may be a confounding factor. RV94 DPI inhalation therapy also demonstrated an antibacterial effect that was statistically significant (*p* = 0.02; Mann–Whitney test) when compared to the control animals that were dosed 24 h after intranasal MRSA infection in rats (Figure 5). The treatment group had an average 1.2 Log_10_CFU/lungset reduction in pulmonary MRSA titer relative to the average titer in the control rats after receiving a single delivered dose equal to 8.3 mg/kg of RV94 DPI. Together with the pre-dosing study, these data validate in vivo efficacy of RV94 DPI after a single inhaled dose delivered either prophylactically or therapeutically against a virulent strain of MRSA (USA300) in a rat acute pulmonary infection model.

A noteworthy limitation of this study involves the use of an acute model of pulmonary MRSA infection to investigate the efficacy of the novel therapy when the proposed indication represents an unmet need that is chronic and difficult to treat [11]. To achieve initial proof of concept and for reasons discussed above, the model described in this report was the best option. However, in future work, a model that more accurately represents CF MRSA lung disease, where a chronic dosing paradigm could be investigated, would be desirable. Moreover, while the benefits of local delivery can be appreciated on the basis of reducing the potential for renal toxicity observed with systemically administered MRSA therapies such as vancomycin and telavancin [5,18], it is unknown at this time whether local toxicities could persist from chronic dosing of RV94 DPI; follow up studies would have to be conducted to better understand how this drug product would perform in repeat dosing regimen.

## 5. Conclusions

There are currently no approved therapies for the treatment of chronic pulmonary MRSA infection in CF. This is problematic since approximately 25% of the patient population is affected and it has been shown to negatively impact survival. RV94 is a next generation lipoglycopeptide that is significantly more potent than vancomycin against planktonic, intracellular, and biofilm MRSA in preclinical models. A spray-dried high-dose DPI of the investigational compound RV94 formulated with trileucine that contains a drug mass concentration in the range of 85–90% has been developed with the goal of potentially enhancing patient convenience. RV94 DPI possesses preclinical PK characteristics when administered as a single inhaled dose to rats that is supportive of infrequent administration with a calculated half-life of 3.5 days. Interestingly, RV94 DPI demonstrated improved lung clearance when compared to a nebulized inhaled single dose of the same compound, and this may be explained by better dispersion and/or dissolution of the therapeutic in the lung tissue when administered as a pharmaceutical powder for inhalation. Furthermore, only trace levels of RV94 were detected in the systemic circulation, which potentially reduces the probability of unwanted side effects (e.g., nephrotoxicity) that have been reported from parenteral lipoglycopeptide therapy. In vivo efficacy was established using both pre- and post-challenge single dose administrations of RV94 DPI in a rat acute pulmonary MRSA infection model and resulted in a statistically significant reduction in lung MRSA titer for both dosing paradigms. While these preclinical data are supportive of the potential for clinical development of an inhaled product targeting pulmonary MRSA infections, the RV94 DPI product has not entered clinical development.

## Figures and Tables

**Table 1:** In vitro aerosol performance of RV94 DPI; *n* = 3. MMAD = mass median aerodynamic diameter determined from investigation using a next generation impactor. GSD = geometric standard deviation. FPF = fine particle fraction.

	MMAD (μm)	GSD	FPF (%)
Average	1.96	2.10	73.4
Standard Deviation	0.15	0.02	3.0

**Table 2:** MIC range of RV94, RV94 DPI, and vancomycin versus *S. aureus*, using CLSI testing protocol. Methicillin-susceptible *S. aureus* (MSSA) = ATCC 29213, methicillin-resistant *S. aureus* (MRSA) = ATCC BAA-1556 (USA300); *n* = 8 for RV94 MRSA and MSSA MICs, *n* = 1 for RV94 DPI MIC versus MRSA and MSSA, *n* = 9 for vancomycin MRSA MIC, and *n* = 6 for vancomycin MSSA MIC.

	MSSA MIC (µg/mL)	MRSA MIC (µg/mL)
RV94	0.031–0.063	0.031–0.063
RV94 DPI	0.063	0.063
Vancomycin	0.5–1	0.5–2

**Table 3:** Summary of PK parameters calculated for RV94 DPI administered by nose-only inhalation to healthy rats, using a VAG dry powder dispenser. Animals were dosed in *n* = 3 cohorts with an average delivered dose of RV94 equal to 4.0 ± 0.6 mg/kg; *n* = 5 animals for immediately post-dose (approximately 0.5 h after dose completion), and *n* = 3 for all other timepoints. LOD for lung based on the lowest standard curve standard was 0.3 μg/g. LOQ = 0.0015 μg/mL for plasma. Data were fit using a one-phase decay in GraphPad Prism 9.3. R^2^ = 0.85 for lung RV94 and 0.68 for plasma RV94.

Delivered Dose (mg/kg)	Tissue	Cmax (μg/g) or (μg/mL)	Tmax (h)	t_1/2_ (Day)	Plateau (μg/g) or (μg/mL)	AUC_0–21Day_ (μg*Day/g) or (μg*Day/mL)
4.0	Lung	126.6	3	3.5	17.6	787.7
	Plasma	0.141	3	1.3	0.004	0.31

## Supplementary Materials

The following supporting information can be downloaded at https://www.mdpi.com/article/10.3390/pharmaceutics15092250/s1. Figure S1: Geometric particle size distribution of RV94 DPI at t = 0 (green curve) and t = 1 month (red curve) measured using laser diffraction; Figure S2: X-ray powder diffraction (XRPD) patterns of RV94-DPI at t = 0 and t = 1 month when stored in a desiccator at room temperature; Figure S3: RV94-DPI deposition on NGI stages based on the emitted dose; Table S1: X-ray photoelectron spectroscopy (XPS) of RV94 DPI.
