# Supplementary material for: Preclinical Investigation of a Lipoglycopeptide Dry Powder Inhalation Therapy for the Treatment of Pulmonary MRSA Infection
Source: Pharmaceutics. 2023 Aug 31;15(9):2250. doi: 10.3390/pharmaceutics15092250 (PMC10537538; doi:10.3390/pharmaceutics15092250)
Supplement: Supplementary file 1 [file pharmaceutics-15-02250-s001.zip › pharmaceutics-2475050-supplementary.pdf]

Supplementary Materials: Preclinical Investigation of a Lipoglycopeptide Dry Powder Inhalation Therapy for the Treatment of Pulmonary MRSA Infection

Authors: Donna M. Konicek#, Adam J. Plaunt, Sachin Gharse, Sasha J. Rose, Arielle Dorfman, Amruta Sabnis, Thomas Baker, Helena Gauani, Donald Chun, Zhili Li, Walter R. Perkins, David Cipolla, and Vladimir S. Malinin

Insmed Incorporated, Bridgewater, New Jersey, USA

# Address correspondence to Donna M. Konicek, donna.konicek@insmed.com

METHODS

X-ray photoelectron spectroscopy (XPS). XPS was used to determine the proportions of the RV94 and trileucine on the surface of the spray dried particles (Rutgers University K-Alpha XPS Facility). The powder was mounted on carbon tape and charge neutralization was performed using 2 eV Ar+ ions. XPS analysis was performed using the K-alpha XPS system (Thermo Scientific, Waltham, MA) with the following parameters: x-ray source was Al Kα (monochromated), x-ray energy was 1486.6 eV, resolution was 0.5 eV, pass energy was 50 eV, step size was 0.1 eV, dwell time was 50 ms, and spot size was 400 µm.

Particle Size Distribution Determined by Laser Diffraction. Laser diffraction was employed to measure the geometric size and distribution of particles in the dry powders using a Sympatec HELOS/BR unit equipped with an ASPIROS feeder and a RODOS/M dry powder dispersing unit (Sympatec GmbH, Clausthal-Zellerfeld, Germany). Approximately 5 mg of the dry powder was added to the sample vial, which was then inserted into the ASPIROS feeder. The sample was measured at measuring range R1 with a RODOS standard trigger at a primary pressure of 0.5 bar and feed velocity of 15 mm/sec.

X-ray powder diffraction (XRPD). X-ray powder diffraction of RV94 DPI was analyzed at the Rutgers University Center for Ceramic Research using a PANalytical X'Pert Diffractometer (Malvern, Worcestershire, United Kingdom). The powder was packed in a zero-background sample holder and XRD was employed at 45kV and 40 mA with Cu Kα (λ= 1.540598 Å) radiation at a scanning rate of 0.04 rad degrees per min. The

scanning range was between 4° to 40° degrees (2θ) with a time per step of 97.92 seconds and a step size of 0.0131°.

***In vitro* Aerosol Characterization.** Aerodynamic Particle Size Distribution (APSD) characterization was performed using the Next Generation Impactor (NGI) (MSP Corp., Shoreview, MN) assembled in-line with the USP (United States Pharmacopeia) induction port, pre-separator, and filter assembly and placed into an environmental control chamber equilibrated to 23° C and 35% relative humidity. Approximately 10 mg of RV94 DPI was added to a size 3 HPMC capsule (Qualicaps Inc., Whitsett, NC) and the capsule was loaded into the mono-dose RS01 high resistance dry powder inhaler from Plastiape (Plastiape S.p.A., Osnago, Italy). The loaded device was then inserted into the USP induction port via an adapter and a vacuum pump along with the TPK 2100 Critical Flow Controller (MSP Corp., Shoreview, MN) to maintain the desired flow rate of 60 L/min for 4 s to aerosolize the dry powder. Following deposition of aerosol within the NGI, aerosol recovery from each component was performed by adding a specified volume of solvent to the NGI collection cups (5 mL), induction port and adapter (10 mL), pre-separator (10 mL), filter (5 mL), device (10 mL), and aerosolized capsule (10 mL). The mass of RV94 associated with each component was quantified using an HPLC system and charged aerosol detector (CAD) (Thermo Fisher Scientific, Waltham, MA) on a C18 column. The emitted dose represents the mass of RV94 that exited the device and was recovered from the NGI components. The aerosol MMAD was calculated in accordance with compendia [1, 2] utilizing cut-off diameters of 8.06, 4.46, 2.82, 1.66, 0.94, 0.55, and 0.34 µm for stages 1 to 7, respectively. The FPF represents the proportion of RV94 that is present in particles with an aerodynamic diameter less than 5 µm and is expressed relative to the total emitted dose.

RESULTS

API	Excipient	Theoretical weight ratio in the powder (RV94/Trileucine)	Measured weight ratio on surface (RV94/Trileucine)
RV94	Trileucine	87.5/12.5	67.7/32.3

Table S1. X-ray photoelectron spectroscopy (XPS) of RV94 DPI. The data demonstrate that trileucine is preferentially deposited on the surface of RV94 DPI compared to its weight ratio in the powder. The higher surface deposition of trileucine could play an important role in the improved aerodynamic properties of the powder, potentially enhancing the stability and aerosol performance of the powder [3, 4].

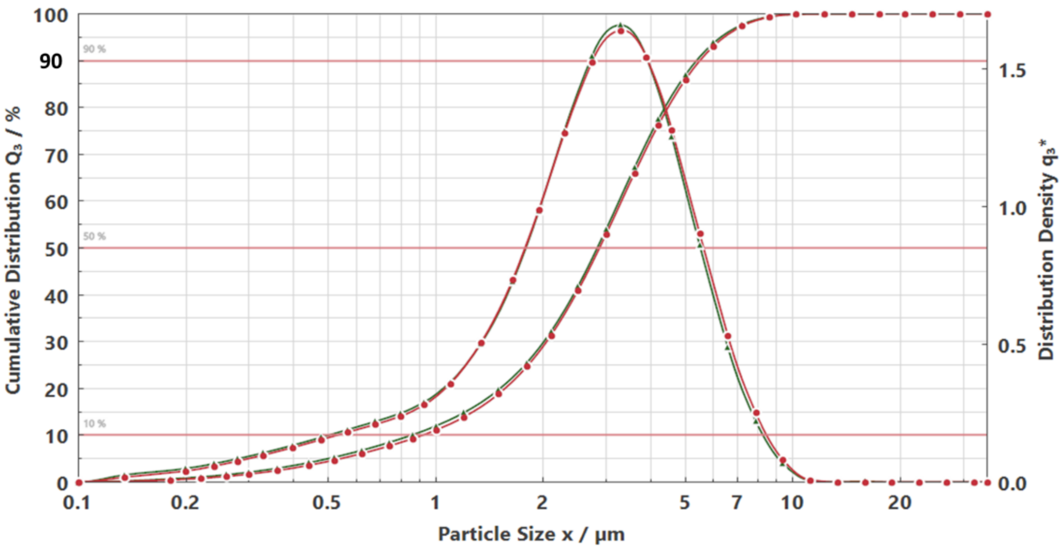

Figure S1. Geometric particle size distribution of RV94 DPI at t=0 (green curve) and t=1 month (red curve) measured using laser diffraction. The powder displayed a unimodal particle size distribution with a mean diameter (d_{50}) equal to 2.8 µm. The particle size distribution did not change after 1 month of storage in a desiccator at room temperature.

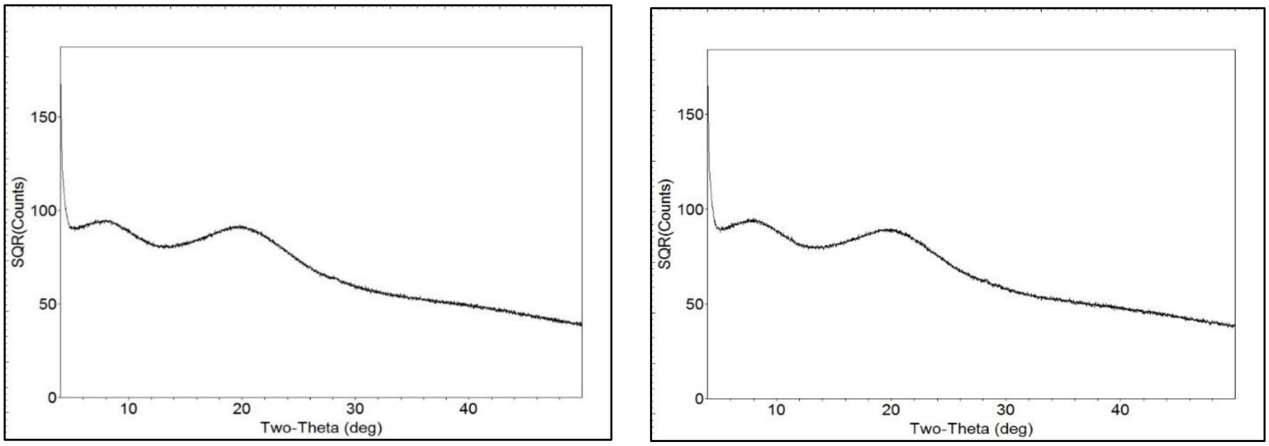

Figure S2. X-ray powder diffraction (XRPD) patterns of RV94-DPI at t=0 (left) and t=1 month in a desiccator at room temperature (right). Analysis was performed on RV94 DPI to investigate the solid-state properties of the powder in order to determine its degree of crystallinity. RV94 DPI displayed a non-crystalline, amorphous pattern both after manufacture and after 1 month of storage in a desiccator at room temperature, indicating that there was no measurable change in the solid-state properties of the powder.

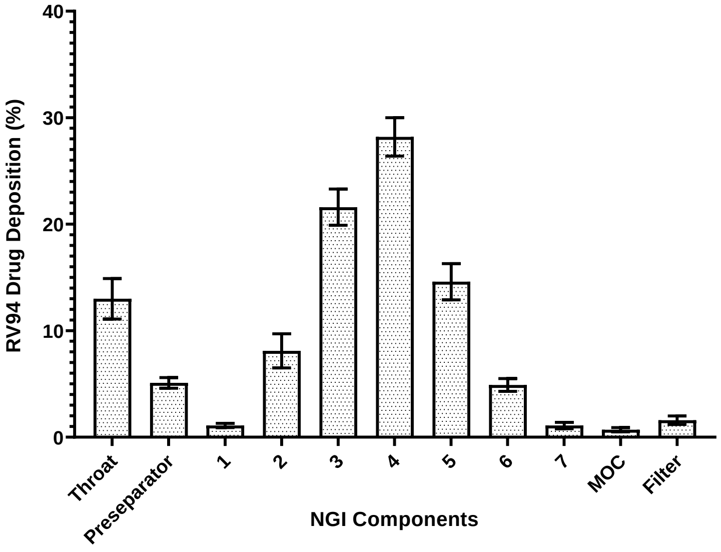

Figure S3. RV94-DPI deposition on NGI stages based on the emitted dose (n=3; data plotted as mean, error is standard deviation). At a flow rate of 60 L/min, majority of the emitted particles were deposited on NGI stages 2-5 with mean aerodynamic diameter cut-off values of 0.94 µm to 4.46 µm.

References

1. *United States Pharmacopeia and National Formulary (USP 43-NF 38), <601> Inhalation and Nasal Drug Products: Aerosols, Sprays, and Powders-Performance Quality Tests, 2012*.
2. *European Pharmacopoeia, 2.9.18. Preparations for Inhalation: Aerodynamic Assessment of Fine Particles, Monograph 20918, 2008*.
3. Shetty, N., et al., *Physical stability of dry powder inhaler formulations.* Expert Opin Drug Deliv, 2020. **17**(1): p. 77-96.
4. Lechuga-Ballesteros, D., et al., *Trileucine improves aerosol performance and stability of spray-dried powders for inhalation.* J Pharm Sci, 2008. **97**(1): p. 287-302.
